# Risk factors associated with the occurrence of the adverse event phlebitis in hospitalized adult patients

**DOI:** 10.1590/0034-7167-2024-0162

**Published:** 2024-10-07

**Authors:** Maryana da Silva Furlan, Amanda Saba, Antônio Fernandes Costa Lima

**Affiliations:** I Universidade de São Paulo. São Paulo, São Paulo, Brazil

**Keywords:** Risk Factors, Phlebitis, Inpatients, Safety Management, Nursing Care, Factores de Riesgo, Flebitis, Pacientes Internos, Administración de la Seguridad, Atención de Enfermería

## Abstract

**Objectives:**

to synthesize knowledge regarding risk factors associated with occurrence of adverse event phlebitis in hospitalized adult patients.

**Methods:**

an integrative literature review, carried out in the CINAHL, PubMed, Virtual Health Library, Embase, Web of Science and Scopus databases. The stages were carried out independently by two reviewers, and the data were analyzed descriptively.

**Results:**

from the analysis of 31 quantitative primary studies, the following risk factors were summarized: length of stay; use of antibiotics; peripheral intravenous catheter dwell time; receive less nursing care; catheter inserted multiple times; patients with infection and comorbidities; presence of pain at catheter insertion site; Teflon® catheter use; reduced patient mobility; quality of patient’s vein; skin elasticity; unsuccessful insertion.

**Conclusions:**

it is necessary to standardize the format for measuring occurrence of this adverse event and develop new studies with a higher level of evidence.

## RESUMEN

**Objetivos:**

sintetizar el conocimiento sobre los factores de riesgo asociados a la aparición del evento adverso flebitis en pacientes adultos hospitalizados.

**Métodos:**

revisión integrativa de la literatura, realizada en las bases de datos CINAHL, PubMed, Biblioteca Virtual en Salud, Embase, *Web of Science* y Scopus. Dos revisores llevaron a cabo los pasos de forma independiente y los datos se analizaron de forma descriptiva.

**Resultados:**

del análisis de 31 estudios primarios cuantitativos se resumieron los siguientes factores de riesgo: duración de la estancia hospitalaria; uso de antibióticos; tiempo de permanencia del catéter intravenoso periférico; recibir menos atención de enfermería; catéter insertado varias veces; pacientes con infección y comorbilidades; presencia de dolor en el sitio de inserción del catéter; uso de catéter de Teflon®; movilidad reducida del paciente; calidad de la vena del paciente; elasticidad de la piel; inserción fallida.

**Conclusiones:**

es necesario estandarizar el formato de medición de la ocurrencia de este evento adverso y desarrollar nuevos estudios con mayor nivel de evidencia.

## INTRODUCTION

In the healthcare area, patient safety consists of a set of activities that create processes, cultures, procedures, behaviors and environments aimed at reducing risks, sustainably and continuously, minimizing occurrence of avoidable damage and errors and, consequently, its impacts^(1)^.

Quality of care in hospital settings is related to the quality of care provided by professionals who carry out their activities in this location^(2)^. Some adverse events (AE) have drawn attention due to the frequency in which they occur, indicating that it is necessary to understand their magnitude in order to implement effective risk management measures to prevent new AEs^(3)^.

Failures resulting from intravenous therapy (IVT) include infiltration, catheter lumen occlusion, local infection, bloodstream infection and phlebitis. Phlebitis is characterized as inflammation of the tunica intima, and its clinical manifestations include pain, edema, erythema, thrombosis and palpable cord^(4)^. Its etiology may have chemical, mechanical, infectious or post-infusional origin^(5)^. Peripheral intravenous catheter (PIC) dwell time, puncture site and/or anatomical region, patient length of stay, number of accesses, female gender, IVT with antibiotics, PIC intermittent maintenance and insertion in the emergency room are risk factors related to the development of phlebitis^(6)^.

Knowing risk factors associated with occurrence of AE phlebitis in patients using PIC allows the development of barriers to avoid complications related to the use of this device during IVT, contributing to safe practice and providing evidence for decision-making in patient care and risk reduction.

Therefore, by instituting a continuous approach to minimize the risks associated with healthcare, institutions are proactively protecting patient safety^(7)^. Despite advances in studies^(6, 8)^ that identified risk factors for phlebitis, it is clear that there is still no consensus, and it is necessary to assess risk factors in randomized clinical trials^(8)^.

In Brazil, an integrative review carried out from 2004 to April 2015 showed that there are still controversies regarding risk factors for phlebitis related to using PIC, and indicated the need to develop research with a strong level of evidence, deepening investigations into its etiology and associated factors^(6)^. Considering the potential of a new integrative review to assess the state of the art regarding verticalization of knowledge and identify new factors associated with occurrence of AE phlebitis, the present study was conducted.

## OBJECTIVES

To synthesize knowledge regarding risk factors associated with occurrence of AE phlebitis in hospitalized adult patients.

## METHODS

### Ethical aspects

Considering article design (review), there was no need for approval from a Research Ethics Committee.

### Study design, location and data collection period

This is an integrative review conducted from December 2022 to February 2023 to answer the guiding question: what are the risk factors for occurrence of phlebitis in adult patients hospitalized with PIC? To this end, the PICO strategy was used, an acronym for Population, Intervention, Comparison and Outcomes^(9, 10)^, considering the letters and their equivalent terms: “P” - adult patients hospitalized with PIC; “I” - risk factors; “C” - no intervention was established for comparison; and “O” - occurrence of phlebitis.

The following steps were taken: 1) research question elaboration; 2) literature search; 3) definition of the information to be extracted from articles; 4) critical assessment of evidence included; 5) interpretation of results; 6) synthesis of knowledge and data presentation^(11)^.

### Sample, inclusion and exclusion criteria

Original studies, available online in full, published from 2015 to December 2022 - this search period is justified to cover the period covered by a previously published integrative review (from 2004 to April 2015)^(6)^ - in Portuguese, English and Spanish, covering adult hospitalized patients who used PIC, were included in the review. Integrative or systematic review studies as well as those conducted in outpatient services were excluded. The option of studies carried out in hospital settings was justified by the fact that patients frequently require, for different clinical-surgical indications, IVT via PIC, increasing the possibility of occurrence of risk factors for the development of phlebitis.

### Study protocol

Data were collected in the Medical Literature Analysis and Retrieval System Online (MEDLINE) via PubMed, Latin American and Caribbean Literature in Health Sciences (LILACS), through the Virtual Health Library (VHL), Excerpta Medica Database (Embase), Cumulative Index to Nursing & Allied Health Literature (CINAHL), SciVerse Scopus (Scopus) and Web of Science databases, establishing controlled descriptors and specific keywords, which were combined with the Boolean operators OR and AND, as described below:

- PubMed: ((“Phlebitis”[MeSH Terms] OR “Phlebitis”[Text Word]) AND (“Risk Factors”[MeSH Terms] OR “Risk Factor”[Text Word]) AND (“catheterization, peripheral”[MeSH Terms] OR “Peripheral Venous Catheterization”[All Fields] OR “Peripheral Venous Catheterizations”[All Fields])) AND (humans[Filter]) - 46 studies;
- VHL: (( mh:(“Phlebitis”)) OR Phlebits) AND (( mh:(“Risk Factors”)) OR “Risk Factors”) AND ((( mh:(“Catheterization, Peripheral”)) OR “Catheterization, Peripheral”) OR (“Peripheral Venous Catheterization” OR “Peripheral Venous Catheterizations”)) - 33 studies;
- CINAHL: ((MH “Phlebitis”) OR “Phlebitis”) AND ((MH “Risk Factors”) OR “Risk Factors”) AND ((MH “Catheterization, Peripheral”) OR “Catheterization, Peripheral” OR “Peripheral Venous Catheterization” OR “Peripheral Venous Catheterizations”) - 49 studies;
- Embase: (‘phlebitis’/exp OR phlebitis) AND (‘risk factor’/exp OR ‘risk factor’) AND (‘peripheral venous catheter’/exp OR ‘peripheral venous catheter’) - 48 studies;
- Web of Science: “Phlebitis”AND (“Peripheral Venous Catheterization” OR “Peripheral Venous Catheterizations”) - 7 studies;
- Scopus: “Phlebitis” AND (“Peripheral Venous Catheterization” OR “Peripheral Venous Catheterizations”) - 19 studies.

The 202 identified articles were grouped and sent to EndNote®, and, after removing duplicate titles, they were imported into Rayyan®, available at https://rayyan.qcri.org for selecting articles included in the sample.

Subsequently, two examiners independently assessed the titles and abstracts, based on the inclusion and exclusion criteria. At each stage of the study selection and eligibility process, disagreements among examiners were resolved by consensus or through the participation of a third examiner.

**Figure 1:**
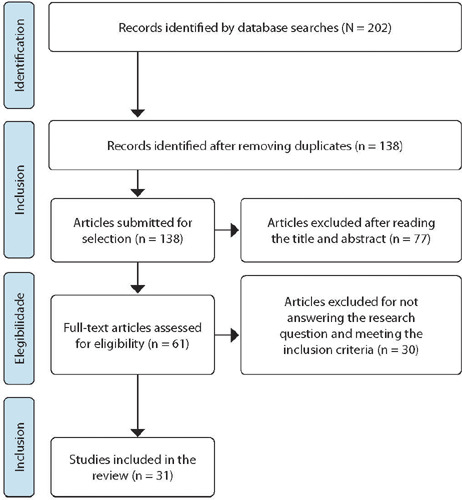
Flow of identification and selection of articles included in the integrative review (n=31) through database searches, São Paulo, SP, Brazil, 2023

In Figure 1, the flowchart for identifying and selecting primary studies was presented in accordance with the Preferred Reporting Items for Systematic Reviews and Meta-Analyses (PRISMA) recommendations^(12)^.

### Data extraction

Data were extracted from sources of evidence by two blind reviewers using a data extraction tool developed by the authors covering article title, year of publication, country of research, study design and risk factors associated with occurrence of phlebitis in hospitalized adult patients.

### Analysis of results

The articles included in the sample were analyzed descriptively and classified according to the level of evidence: level I (evidence from a systematic review or meta-analysis of all randomized controlled clinical trials or from clinical guidelines based on systematic reviews of randomized controlled clinical trials; level II (evidence derived from well-designed randomized controlled trials); level III (evidence obtained from well-designed clinical trials without randomization); systematic review of qualitative and descriptive studies); level VI (evidence derived from a single descriptive or qualitative study); and level VII (evidence derived from the opinion of authorities and/or report from expert committees)^(13)^.

## RESULTS

In this integrative review, 31 primary articles^(14, 15, 16, 17, 18, 19, 20, 21, 22, 23, 24, 25, 26, 27, 28, 29, 30, 31, 32, 33, 34, 35, 36, 37, 38, 39, 40, 41, 42, 43, 44)^ were included, published in 14 countries, as shown in Chart 1. It can be seen that Brazil was the country with the highest number of studies (n=8), followed by Australia (n=3) and Turkey (n=3). The number of studies relating to the years 2016 (n=7) stood out, followed by the years 2015, 2019 and 2022 (n=3). Most of the studies analyzed came from observational cohort studies and randomized clinical trials.

**Chart 1:** Characterization of the 31 primary studies included in the integrative review according to citation, title, design, year and country, São Paulo, São Paulo, Brazil, 2023

Quote	Title	Design	Year	Country
14	Relative incidence of phlebitis associated with peripheral intravenous catheters in the lower versus upper extremities	Prospective cohort study	2015	Israel
15	Incidence and factors associated with the development of phlebitis: results of a pilot cohort study	Prospective cohort study	2015	Colombia
16	Incidence of local complications in peripheral venous catheters and associated risk factors	Prospective cohort study	2015	Brazil
17	Nursing care as a predictor of phlebitis related to insertion of a peripheral venous cannula in emergency departments: findings from a prospective study	Prospective study	2016	Italy
18	The Development of Phlebitis and Infiltration in Patients with Peripheral Intravenous Catheters in the Neurosurgery Clinic and Affecting Factors	Descriptive and cross-sectional study	2016	Turkey
19	Phlebitis associated with peripheral intravenous catheters in adults admitted to hospital in the Western Brazilian Amazon	Exploratory study	2016	Brazil
20	Peripheral intravenous catheters in situ for more than 96 h in adults: What factors affect removal?	Cross-sectional study	2016	China
21	Low-angled peripheral intravenous catheter tip placement decreases phlebitis	Observational prospective	2016	Japan
22	Complications related to the use of peripheral venous catheters: a randomized clinical trial	Randomized clinical trial	2016	Brazil
23	Incidence of phlebitis associated with the use of peripheral IV catheter and following catheter removal	Cohort study	2016	Brazil
24	Incidence of phlebitis and post-infusion phlebitis in hospitalised adults	Cohort study	2017	Brazil
25	Incidence and risk factors of phlebitis associated to peripheral intravenous catheters	Cohort study	2017	Spain
26	Observational Study of Peripheral Intravenous Catheter Outcomes in Adult Hospitalized Patients: A Multivariable Analysis of Peripheral Intravenous Catheter Failure	Prospective cohort	2017	Australia
27	Dressings and securements for the prevention of peripheral intravenous catheter failure in adults (SAVE): a pragmatic, randomised controlled, superiority trial	Randomized clinical trial	2018	Australia
28	Incidence, severity and risk factors of peripheral intravenous cannula-induced complications: An observational prospective study	Prospective observational study	2018	Serbia
29	Prevalence of peripheral intravenous catheter-related phlebitis: associated factors	Prospective cohort study	2018	Portugal
30	Phlebitis and infiltration: vascular trauma associated with the peripheral venous catheter	Descriptive cohort study	2018	Portugal
31	Complicações locais da terapia intravenosa periférica e fatores associados	Cross-sectional study	2018	Brazil
32	Phlebitis-related peripheral venous catheterization and the associated risk factors	Prospective observational study	2018	Turkey
33	Use of infrared thermography in the detection of superficial phlebitis in adult intensive care unit patients: A prospective single-center observational study	Prospective observational study	2019	Netherlands
34	A Model of Phlebitis Associated with Peripheral Intravenous Catheters in Orthopedic Inpatients	Prospective study	2019	South Korea
35	The RESPECT trial-Replacement of peripheral intravenous catheters according to clinical reasons or every 96 hours: A randomized, controlled, non-inferiority trial	Multicenter, unblinded, controlled randomized trial	2020	Brazil
36	In-line filtration reduced phlebitis associated with peripheral venous cannulation: Focus on cost-effectiveness and patients’ perspectives	Economic analysis study; randomized clinical trial; cost-effectiveness	2020	Italy
37	Evaluation of risk factors on time to phlebitis- and nonphlebitis-related failure when peripheral venous catheters were replaced as clinically indicated	Prospective cohort study	2020	Turkey
38	Inherent and modifiable risk factors for peripheral venous catheter failure during cancer treatment: a prospective cohort study	Prospective cohort study	2021	Australia
39	Compare the efficacy of recommended peripheral intravascular cannula insertion practices with a standard protocol: A randomized control trial	Randomized clinical trial	2022	India

40	Incidence, risk factors and medical cost of peripheral intravenous catheter-related complications in hospitalised adult patients	Prospective observational study	2022	China
41	Risk factors for peripheral intravenous catheter-related phlebitis in adult patients	Post hoc analysis of a randomized controlled clinical trial	2022	Brazil
42	A Prospective Sonographic Evaluation of Peripheral Intravenous Catheter Associated thrombophlebitis	Prospective cohort study	2022	USA
43	Risk factors for peripheral intravascular catheter-related phlebitis in critically ill patients: analysis of 3429 catheters from 23 Japanese intensive care units	Prospective cohort study	2022	Japan
44	Observational study of peripheral intravenous catheter outcomes in an internal medicine department	Prospective observational study	2022	Israel

From the results in Charts 2, 3 and 4, the prevalence of studies with level of evidence IV (67.7%) is indicated. Among the main outcomes, catheter dwell time, use of antibiotics and other drugs, contrast, catheter material used, gauge and catheter number inserted stood out. There was variability in the way phlebitis was measured, with the application of different instruments. Some studies used the signs of phlebitis themselves to make the diagnosis.

**Chart 2:** Summary table of primary studies included in the integrative review according to risk factors associated with phlebitis relative to the patient and level of evidence, São Paulo, São Paulo, Brazil, 2023

Risk factors associated with occurrence of phlebitis: related to patient	Level of evidence
The presence of phlebitis was statistically significant when associated with female patients^(26)^.	IV
Age (18 to 47 years), patients with more than six days of hospitalization and platelet count (>303,000)^(15)^.	IV
There was statistical significance regarding skin color (white and brown skin associated with grade I phlebitis and black skin associated with grade III phlebitis) and age (the group of patients aged 19 to 48 years and the group aged 71 to 95 years were associated with grade I phlebitis and group of patients aged 49 to 70 years were associated with grade III phlebitis)^(24)^.	IV
The presence of infections and comorbidities increased the chance of developing phlebitis by approximately 1.5 times. Grade III and IV phlebitis were diagnosed in patients over 70 years of age, malnourished and with diabetes *mellitus* ^(28)^.	IV
PICs were less likely to experience phlebitis with each increase in age (per decade) if the patient had three or more comorbidities^(38)^.	IV
Presence of infection and chronic diseases had a significant relationship with the development of phlebitis^(19)^.	IV
Phlebitis was significantly more present in PIC in patients with comorbidities, current infections and with an indwelling urinary catheter^(28)^.	IV
Poor skin elasticity (hazard ratio: 1.47; 95% Confidence Interval: 1.07–2.02; p-value = 0.015)^(37)^.	IV
There was a significant relationship between having a chronic disease and the development of phlebitis^(32)^.	IV
Phlebitis was associated with active patients and vein quality (fair and poor)^(34)^.	IV
Admission to the neurological clinic (p-value 0.018) was compared with admission to the medical clinic^(31)^.	VI
Patients in neurosurgery clinic. There was a greater number of phlebitis in patients with cranial diseases and PIC^(18)^.	VI
Patients in surgical units (may be associated with hypovolemia and long period of PIC use)^(40)^.	IV
The addition of one day, for any period of hospitalization, increased the probability of patients experiencing phlebitis by 1.07 times^(30)^.	IV
Reduced mobility, family history of deep vein thrombosis^(41)^.	II

*PIC - peripheral intravenous catheter.*

**Chart 3:** Summary table of primary studies included in the integrative review according to risk factors associated with phlebitis related to intravenous therapy and level of evidence, São Paulo, São Paulo, Brazil, 2023

Risk factors associated with occurrence of phlebitis: related to IVT	Level of evidence
PIC dwell time greater than 72 hours^(16)^.	IV
PIC dwell time ≥72 hours, forearm puncture and infusion of medications such as ceftriaxone (sample = 7; 25%), clarithromycin (sample = 7; 28%) and oxacillin (sample = 6; 46.2%)^(23)^.	IV
Catheter dwell time of 49-72 hours^(18)^.	VI
PIC dwell time ≥ 3.25 days^(29)^.	IV
There was a statistically significant relationship between the duration of catheterization (49-72 hours), the type of fluid used (isotonic) and the development of phlebitis^(32)^.	IV
PIC dwell time (91.5 hours) was associated with occurrence of phlebitis^(33)^.	IV
Catheter dwell time (25-48 hours), infusion method (continuous and intermittent), use of contrast medium, medications with high osmolarity^(34)^.	IV
It was found that 9.0% of patients developed phlebitis with no difference between groups. PIC replacement, when clinically indicated, was non-inferior to routine replacement (96 hours) in terms of occurrence of phlebitis, and was associated with significantly less phlebitis/1,000 catheter days^(35)^.	II
Administration of less than three medications, administration of mucosal protectors or gastric antisecretory agents and the use of antibacterial medications^(15)^.	IV
There was a significant relationship between phlebitis and type of infusion, and the associated use of continuous and intermittent infusion had a greater relationship with occurrence of phlebitis^(19)^.	VI
PIC-related phlebitis can be significantly associated with intravenous medication (amino acid infusion), with earlier removal anticipated by the insertion site (elbow joint)^(20)^.	IV
Regarding the individual analysis of medication use, tramadol hydrochloride, amoxicillin + clavulanic acid and amphotericin (post-infusional phlebitis) were statistically significant. In the analysis of the association of drug classes, antifungals, antiinflammatories and those that act on the blood and blood-forming organs (which drugs were not specified) showed a positive association^(24)^.	IV
The use of an extension tube as a PIC accessory^(25)^.	IV
Patients using intravenous flucloxacillin or with frequent PIC^(26)^.	IV
Patients with administration, via PIC, of fewer medications (all groups) and risk solutions, with fewer days of administration of groups of medications, with and without risk, and risk solutions^(28)^.	IV
Using in-line filter reduced the incidence of postoperative phlebitis related to PIC and has the potential to contribute to increased patient satisfaction and reduced catheter-related discomfort^(36)^.	II
Use of drugs that irritate the vascular endothelium^(40)^.	IV
PIC on the back of the hand and report of pain, intravenous administration with amoxicillin-clavulanate potassium and omeprazole sodium^(41)^.	II
Use of nitroglycerin (p-value 0.04), nicardipine (p-value 0.0008), noradrenaline (p-value 0.002), amiodarone (p-value 0.0006) and levetiracetam (p-value < 0.0001)^(43)^.	IV
Use of intravenous antibiotics^(44)^.	IV

*IVT- intravenous therapy; PIC - peripheral intravenous catheter.*

**Chart 4:** Summary table of primary studies included in the integrative review according to risk factors associated with phlebitis related to the procedure and level of evidence, São Paulo, São Paulo, Brazil, 2023

Risk factors associated with occurrence of phlebitis: related to the procedure	Level of evidence
Receiving less nursing care was a predictor of PIC-related phlebitis. For every unit increase in missed care, there was a 3.8% increase in the risk of developing phlebitis. The lack of assessment of PIC insertion site and other nursing care delayed the recognition of phlebitis. Being in a specialized hospital was associated with a lower risk of PIC-related phlebitis^(17)^.	IV
Higher number of phlebitis in patients who had PIC inserted in the same location several times and inserted by professionals with four years of training compared to those who had two years of training^(18)^.	VI
Phlebitis occurred more frequently when the catheter tip was placed at an angle >5.8°^(21)^.	IV
Phlebitis was statistically significant when associated with hematomas at the PIC insertion site and insertion on patients’ dominant side^(26)^.	IV
No significant differences in the incidence of dislocation and phlebitis were identified between any of the intervention groups compared to the polyurethane control group^(27)^.	II
Phlebitis was more present in 20- and 18-gauge PICs, made of Teflon®, which were in situ for more than 96 hours. The chance is about twice as large in the 22- and 20-gauge PIC diameter, and is almost three times as high in the 18-gauge PIC^(28)^.	IV
By increasing a PIC to any amount, the probability of a patient developing phlebitis increased by 1.37 times^(30)^.	IV
Multivariate analysis identified that the difference in skin temperature (0.90°) is associated with occurrence of phlebitis^(33)^.	IV
Hand hygiene duration (<10 seconds), clinical nursing experience period (1-3 years)^(34)^.	IV
There was unsuccessful insertion on the first attempt (risk ratio: 1.35; 95% Confidence Interval: 1.04-1.83; p-value = 0.047) and use of locally manufactured catheters (Teflon® manufactured in Turkey) (rate risk: 1.61; 95% Confidence Interval: 1.18–2.20; p-value = 0.002)^(37)^.	IV
Insertion into the wrist joint. There was a significant decrease in the rate of thrombophlebitis after applying the standard insertion site cleaning protocol compared to the Centers for Disease Control and Prevention recommendation. The incidence of thrombophlebitis increased significantly upon the second attempt at PIC insertion^(39)^.	II
The increase in the proportion of the catheter in relation to the size of the vein (greater than or equal to 33.3%) and the steeper angle of the catheter tip (≥5 degrees) increased the risk of thrombophlebitis^(42)^.	IV

*PIC - peripheral intravenous catheter.*

## DISCUSSION

In recent years, there has been an increase in the number of studies on risk factors associated with occurrence of phlebitis in hospitalized adult patients who used PIC. In these studies, variability was found in the way occurrence of this AE was measured. It is noteworthy that, of the 71 phlebitis scales in use around the world, only three have undergone any psychometric analysis, and the lack of consensus on the use of assessment instruments may contribute to the disparities found in the literature^(45)^. Another complicating factor is that some studies^(16, 22, 28, 31, 37, 38, 40)^ analyzed occurrence of phlebitis, infiltration, extravasation and occlusion together, and risk factors were, in general, related to failures related to PIC, consisting of variables that can generate confusion between the risk factors.

Although studies^(14, 18, 23, 28, 29, 32, 34)^ indicate that the dwell time PIC is in place is a risk factor for phlebitis, a systematic review^(46)^, when evaluating the effects of changing the PIC, when clinically indicated, compared with the routinely scheduled change, based on a sample of seven trials randomized clinical trials with 4,895 patients, found that there was no difference between these groups in any of the measures analyzed and that average costs were lower when PICs were replaced according to clinical indication. The study also indicated that routine PIC exchange can cause pain, discomfort and patient dissatisfaction due to new punctures^(46)^.

The catheter material used may be associated with phlebitis^(23, 28, 37)^. The polyurethane device has greater flexibility when compared to Teflon®, minimizing damage caused to the vascular wall and playing an important role in minimizing the development of this AE^(21)^. Flexible catheters, such as polyurethane catheters, are associated with fewer complications and a reduction in phlebitis^(47)^. Furthermore, it is recommended to assess the vessel so that the catheter used is of the appropriate size^(5)^. PICs with a gauge greater than 20 are more associated with occurrence of phlebitis^(5, 25, 28)^.

It is important to consider patients with difficult intravenous access (DIVA), with two or more unsuccessful attempts at peripheral intravenous puncture, using the traditional technique, in addition to the absence of visible or palpable veins or patients with a reported or documented history of DIVA^(48)^. These patients need to be assessed, before puncture, to avoid multiple attempts, as the number of attempts is related to PIC failures^(18, 28, 30, 37, 39)^.

By recognizing that unsuccessful attempts cause harm, the Infusion Nurses Society (INS) recommends that, after two unsuccessful attempts, the procedure should be directed to a practitioner with a higher skill level or the use of alternative routes of administration^(5)^.

The present review highlighted the association of some drugs with occurrence of phlebitis^(18, 23, 26, 27, 29, 37, 41, 43, 44, 46, 47)^, such as ceftriaxone, clindamycin, oxacillin, contrast, nitroglycerin, nicardipine, norepinephrine, amiodarone, amphotericin and tramadol hydrochloride. In this regard, it is emphasized that safe IVT planning, minimizing possible risks, cannot do without adequate knowledge of drugs that are harmful to the venous network^(49)^.

A Spanish study, carried out by a team of doctors, nurses and pharmacists, standardized the dilutions of the non-oncological medications most used in hospitalized adult patients. The list of medications was established through consensus with the aim of reducing variability in care, improving safety in IVT and suggesting different levels of risk. Knowing the medications and their characteristics contributes to choosing the most appropriate device for patients, aiming to reduce occurrence of complications such as phlebitis^(49)^.

Nursing care is essential for managing PIC and minimizing occurrence of AEs; missed nursing care affects the incidence of phlebitis^(34)^; the lack of assessment of PIC insertion site delays the recognition of phlebitis^(17)^. Furthermore, during assessment, patients may report the presence of pain, a symptom that may be associated with phlebitis^(41)^.

The presence of infection^(19, 28)^ and chronic diseases^(19, 28, 32, 38)^ was present as a risk factor for phlebitis. Therefore, nurses need to know them to provide individualized care to these patients, who may remain hospitalized for a longer period, and prolonged hospitalization increases the risk of developing phlebitis^(15, 30)^.

The results obtained in this review are similar to those previously mentioned^(6)^, notably in relation to the diversity in the way phlebitis is measured, which indicates the need for further investigations into the great variability of the instruments used, requiring alignment between the researchers on this topic. Other factors such as PIC dwell time, puncture site, patient length of stay, number of accesses and use of antibiotics were similar to those in the aforementioned review.

It is noteworthy that this review advances in relation to existing knowledge, by highlighting that receiving less nursing care, reduced patient mobility, family history of deep vein thrombosis, report of pain during PIC insertion, quality of patient’s vein, skin elasticity and unsuccessful PIC insertion are associated with occurrence of phlebitis.

Aiming to support decision-making, prior to peripheral intravenous puncture, in order to contribute to preservation of patients’ vascular health, it is corroborated, as an aspect to be considered in future research, the identification of patients with DIVA and its association with occurrence of phlebitis^(50)^. It should be noted that, after two unsuccessful attempts to obtain access via PIC, INS recommends that a professional with a higher level of skill and technological support be called to perform the procedure, in addition to considering another route for administering the drug^(5)^. Therefore, it is recommended to conduct studies with a higher level of evidence that assess patients’ vascular health, the implementation of specific training programs by nurses, the individualization of the care provided, ultrasound-guided peripheral venous puncture and associated outcomes, and the early identification of patients with DIVA, enabling, in addition to avoiding complications, the development of clinical guidelines regarding the care required.

### Study limitations

Only the inclusion of free access articles, available in full in established databases, can be indicated as a limitation of this study.

### Contributions to nursing, health, or public policy

Through this integrative review, risk factors already evidenced in the literature were corroborated, and new factors associated with the development of phlebitis in hospitalized adult patients were identified, notably the recognition of patients with DIVA. From the knowledge generated, the relevance of the proactive approach to preserving vessels for future needs, improving the execution of care plan, reducing hospitalization time and, consequently, costs associated with the care provided stands out.

## CONCLUSIONS

In this integrative review, 31 (100.0%) quantitative primary studies were analyzed on risk factors associated with occurrence of phlebitis in hospitalized adult patients who used PIC, with a predominance of studies with level of evidence IV (67.7%), cohort type (45.1%) and developed in Brazil (25.8%).

The following risk factors were summarized: length of stay; hematological alteration; number of medications administered; use of antibiotics; PIC dwell time; receive less nursing care; catheter inserted multiple times; patients with infection and comorbidities; presence of pain at PIC insertion site; use of Teflon® catheter; reduced patient mobility; family history of deep vein thrombosis; quality of patient’s vein; skin elasticity; and unsuccessful insertion. It is important to emphasize the importance of standardizing the format for measuring occurrence of this AE and developing new studies with a higher level of evidence.
