# Supplementary material for: Risk factors associated with the occurrence of the adverse event phlebitis in hospitalized adult patients
Source: Rev Bras Enferm. 2024 Oct 7;77(5):e20240162. doi: 10.1590/0034-7167-2024-0162 (PMC11458144; doi:10.1590/0034-7167-2024-0162)
Supplement: Translation

# Translation: Fatores de risco associados à ocorrência do evento adverso flebite em pacientes adultos hospitalizados

**DOI:** 10.1590/0034-7167-2024-0162pt

**Authors:** Maryana da Silva Furlan, Amanda Saba, Antônio Fernandes Costa Lima

**Affiliations:** I Universidade de São Paulo. São Paulo, São Paulo, Brasil

**Keywords:** Fatores de Risco, Flebite, Pacientes Internados, Gestão da Segurança, Cuidados de Enfermagem

## Abstract

**Objetivos:**

sintetizar o conhecimento relativo aos fatores de risco associados à ocorrência do evento adverso flebite em pacientes adultos hospitalizados.

**Métodos:**

revisão integrativa da literatura, realizada nas bases de dados CINAHL, PubMed, Biblioteca Virtual em Saúde, Embase, *Web of Science* e Scopus. As etapas foram conduzidas, independentemente, por dois revisores, e os dados foram analisados descritivamente.

**Resultados:**

a partir da análise de 31 estudos primários quantitativos, foram sintetizados os seguintes fatores de risco: tempo de internação; uso de antibióticos; tempo de permanência do cateter intravenoso periférico; receber menos cuidados de enfermagem; cateter inserido várias vezes; pacientes com infecção e comorbidades; presença de dor no local de inserção do cateter; uso do cateter de Teflon®; mobilidade reduzida do paciente; qualidade da veia do paciente; elasticidade da pele; inserção malsucedida.

**Conclusões:**

é necessário uniformizar o formato de medição da ocorrência desse evento adverso e desenvolver novos estudos com maior nível de evidência.

## INTRODUÇÃO

Na área da saúde, a segurança do paciente consiste em um conjunto de atividades que cria processos, culturas, procedimentos, comportamentos e ambientes visando à redução de riscos, de forma sustentável e contínua, minimizando a ocorrência de dano evitável e erros e, consequentemente, seus impactos^(1)^.

A qualidade da assistência no ambiente hospitalar está relacionada à qualidade do cuidado prestado pelos profissionais que desempenham suas atividades neste local^(2)^. Alguns eventos adversos (EA) têm chamado a atenção pela periodicidade em que ocorrem, indicando ser preciso compreender a sua magnitude a fim de implantar medidas eficazes de gerenciamento de risco na prevenção de novos EA^(3)^.

As falhas decorrentes da terapia intravenosa (TIV) abrangem infiltração, oclusão do lúmen do cateter, infecção local, infecção da corrente sanguínea e flebite. A flebite é caracterizada como a inflamação da túnica íntima, e suas manifestações clínicas incluem dor, edema, eritema, trombose e cordão palpável^(4)^. Sua etiologia pode ter origem química, mecânica, infecciosa ou pós-infusional^(5)^. O tempo de permanência do cateter intravenoso periférico (CIP), local de punção e/ou região anatômica, tempo de internação do paciente, quantidade de acessos, sexo feminino, TIV com antibióticos, manutenção intermitente e inserção do CIP na emergência são fatores de risco relacionados ao desenvolvimento da flebite^(6)^.

Conhecer os fatores de risco associados à ocorrência do EA flebite, em pacientes que utilizam CIP, permite desenvolver barreiras para evitar complicações relacionadas ao uso desse dispositivo durante a TIV, contribuindo para a prática segura e fornecendo evidências para tomada de decisão no cuidado aos pacientes e redução dos riscos.

Portanto, ao instituir uma abordagem contínua para minimizar os riscos associados ao cuidado em saúde, as instituições estão protegendo, de forma proativa, a segurança do paciente^(7)^. Apesar dos avanços nos estudos^(6, 8)^ que identificaram os fatores de risco para flebite, é evidenciado que ainda não há consenso, sendo preciso avaliar os fatores de risco em ensaios clínicos randomizados^(8)^.

No Brasil, revisão integrativa realizada de 2004 a abril de 2015 evidenciou que ainda existem controvérsias sobre os fatores de risco para flebite relacionada ao uso de CIP, e indicou a necessidade do desenvolvimento de pesquisas com forte nível de evidência, aprofundando as investigações sobre a sua etiologia e fatores associados^(6)^. Considerando o potencial de uma nova revisão integrativa para avaliar o estado da arte relativo à verticalização do conhecimento e identificar novos fatores associados à ocorrência do EA flebite, conduziu-se o presente estudo.

## OBJETIVOS

Sintetizar o conhecimento relativo aos fatores de risco associados à ocorrência do EA flebite em pacientes adultos hospitalizados.

## MÉTODOS

### Aspectos éticos

Considerando o tipo de artigo (revisão), não houve a necessidade de aprovação de um Comitê de Ética em Pesquisa.

### Desenho, local do estudo e período da coleta de dados

Trata-se de revisão integrativa conduzida, nos meses de dezembro de 2022 a fevereiro de 2023, para responder à questão norteadora: quais são os fatores de risco para a ocorrência de flebite em pacientes adultos hospitalizados com CIP? Para tanto, empregou-se a estratégia PICO, acrônimo de População, Intervenção, Comparação e Outcomes/Desfecho^(9, 10)^, considerando as letras e seus termos equivalentes: “P” - pacientes adultos hospitalizados com CIP; “I” - fatores de risco; “C” - não se estabeleceu intervenção para comparação; e “O” - ocorrência de flebite.

Foram percorridas as seguintes etapas: 1) elaboração da questão de pesquisa; 2) busca na literatura; 3) definição das informações a serem extraídas dos artigos; 4) avaliação crítica das evidências incluídas; 5) interpretação dos resultados; 6) síntese do conhecimento e apresentação dos dados^(11)^.

### Amostra, critérios de inclusão e exclusão

Foram incluídos na revisão estudos originais, disponíveis *online* na íntegra, publicados a partir de 2015 até dezembro de 2022 - justifica-se esse período de buscas para abranger o período coberto por revisão integrativa publicada anteriormente (de 2004 a abril de 2015)^(6)^ - nas línguas portuguesa, inglesa e espanhola, abrangendo pacientes adultos hospitalizados que utilizaram CIP. Excluíram-se estudos de revisão integrativa ou sistemática, bem como os conduzidos em serviços ambulatoriais. Justificou-se a opção dos estudos desenvolvidos em ambiente hospitalar pelo fato de os pacientes requererem frequentemente, por diferentes indicações clínico-cirúrgicas, TIV via CIP, aumentando a possibilidade da ocorrência de fatores de risco para o desenvolvimento de flebite.

### Protocolo de estudo

Os dados foram coletados nas bases de dados *Medical Literature Analysis and Retrieval System Online* (MEDLINE) via PubMed, Literatura Latino-Americana e do Caribe em Ciências da Saúde (LILACS), por meio da Biblioteca Virtual em Saúde (BVS), *Excerpta Medica Database* (Embase), *Cumulative Index to Nursing & Allied Health Literature* (CINAHL), SciVerse Scopus (Scopus) e *Web of Science,* estabelecendo-se os descritores controlados e palavras-chaves específicas, os quais foram combinados com os operadores booleanos *OR* e AND, conforme descrito a seguir:

- PubMed: ((“*Phlebitis”[MeSH Terms] OR “Phlebitis”[Text Word]) AND (“Risk Factors”[MeSH Terms] OR “Risk Factor”[Text Word]) AND (“catheterization, peripheral”[MeSH Terms] OR “Peripheral Venous Catheterization”[AllFields] OR “Peripheral Venous Catheterizations”[All Fields])) AND (humans[Filter])* - 46 estudos;
- BVS: *((mh:(“Phlebitis”)) OR Phlebits) AND ((mh:(“Risk Factors”)) OR “RiskFactors”) AND (((mh:(“Catheterization, Peripheral”)) OR “Catheterization, Peripheral”) OR (“Peripheral Venous Catheterization” OR “Peripheral Venous Catheterizations”))* - 33 estudos;
- CINAHL: *((MH “Phlebitis”) OR “Phlebitis”) AND ((MH “Risk Factors”) OR “Risk Factors”) AND ((MH “Catheterization, Peripheral”) OR “Catheterization, Peripheral” OR “Peripheral Venous Catheterization” OR “Peripheral Venous Catheterizations”) -* 49 estudos;
- Embase: *(‘phlebitis’/exp OR phlebitis) AND (‘risk factor’/exp OR ‘risk factor’) AND (‘peripheral venous catheter’/exp OR ‘peripheral venous catheter’) -* 48 estudos;
- *Web of Science: “Phlebitis” AND (“Peripheral Venous Catheterization” OR “Peripheral Venous Catheterizations”) - sete* estudos;
- Scopus: “*Phlebitis” AND (“Peripheral Venous Catheterization” OR “Peripheral Venous Catheterizations”)* - 19 estudos.

Os 202 artigos identificados foram agrupados e enviados para o *EndNote*®, e, após remoção dos títulos duplicados, foram importados para o aplicativo *web Rayyan*®, disponível em https://rayyan.qcri.org para a seleção dos artigos componentes da amostra.

Posteriormente, dois examinadores avaliaram, de maneira independente, os títulos e os resumos, fundamentados nos critérios de inclusão e de exclusão. Em cada etapa do processo de seleção e elegibilidade dos estudos, resolveram-se as divergências entre os examinadores por consenso ou por intermédio da participação de um terceiro examinador.

Apresentou-se, na Figura 1, o fluxograma de identificação e seleção dos estudos primários de acordo com as recomendações do *Preferred Reporting Items for Systematic Reviews and Meta-A-nalyses* (PRISMA)^(12)^.

**Figura 1:** Fluxo de identificação e seleção dos artigos incluídos na revisão integrativa (n=31) por meio de busca nas bases de dados, São Paulo, São Paulo, Brasil, 2023

### Extração dos dados

Os dados foram extraídos das fontes de evidência por dois revisores cegos usando uma ferramenta de extração de dados elaborada pelos autores abrangendo título do artigo, ano de publicação, país de realização da pesquisa, desenho do estudo e fatores de risco associados à ocorrência de flebite em pacientes adultos hospitalizados.

### Análise dos resultados

Os artigos incluídos na amostra foram analisados descritivamente e classificados de acordo com o nível de evidência: nível I (evidências de revisão sistemática ou metanálise de todos ensaios clínicos randomizados controlados ou oriundos de diretrizes clínicas baseadas em revisões sistemáticas de ensaios clínicos randomizados controlados; nível II (evidências derivadas de ensaios clínicos randomizados controlado bem delineados); nível III (evidências obtidas de ensaios clínicos bem delineados sem randomização); nível IV (evidências provenientes de estudos de coorte e de casos-controle bem delineados); nível V (evidências originárias de revisão sistemática de estudos qualitativos e descritivos); nível VI (evidências derivadas de estudo descritivo ou qualitativo único); e nível VII (evidências oriundas de opinião de autoridades e/ou relatório de comitês de especialistas)^(13)^.

## RESULTADOS

Nesta revisão integrativa, foram incluídos 31 artigos primários^(14, 15, 16, 17, 18, 19, 20, 21, 22, 23, 24, 25, 26, 27, 28, 29, 30, 31, 32, 33, 34, 35, 36, 37, 38, 39, 40, 41, 42, 43, 44)^, publicados em 14 países, conforme apresentado no Quadro 1. Verifica-se que o Brasil foi o país que apresentou o maior número de publicações (n=8), seguido da Austrália (n=3) e Turquia (n=3). Destacaram-se os quantitativos de publicações relativas aos anos de 2016 (n=7), seguidos dos anos 2015, 2019 e 2022 (n=3). A maioria das publicações analisadas foi originada de estudos observacionais do tipo coorte e ensaio clínico randomizado.

**Quadro 1:** Caracterização dos 31 estudos primários incluídos na revisão integrativa segundo citação, título, desenho, ano e país, São Paulo, São Paulo, Brasil, 2023

Citação	Título	Desenho	Ano	País
14	*Relative incidence of phlebitis associated with peripheral intravenous catheters in the lower versus upper extremities*	Estudo de coorte prospectivo	2015	Israel
15	*Incidence and factors associated with the development of phlebitis: results of a pilot cohort study*	Estudo de coorte prospectivo	2015	Colômbia
16	*Incidence of local complications in peripheral venous catheters and associated risk factors*	Estudo de coorte prospectiva	2015	Brasil
17	*Nursing care as a predictor of phlebitis related to insertion of a peripheral venous cannula in emergency departments: findings from a prospective study*	Estudo prospectivo	2016	Itália
18	*The Development of Phlebitis and Infiltration in Patients with Peripheral Intravenous Catheters in the Neurosurgery Clinic and Affecting Factors*	Estudo descritivo e transversal	2016	Turquia
19	*Phlebitis associated with peripheral intravenous catheters in adults admitted to hospital in the Western Brazilian Amazon*	Estudo exploratório	2016	Brasil
20	*Peripheral intravenous catheters in situ for more than 96 h in adults: What factors affect removal?*	Estudo transversal	2016	China
21	*Low-angled peripheral intravenous catheter tip placement decreases phlebitis*	Prospectivo observacional	2016	Japão
22	*Complications related to the use of peripheral venous catheters: a randomized clinical trial*	Ensaio clínico randomizado	2016	Brasil
23	*Incidence of phlebitis associated with the use of peripheral IV catheter and following catheter removal*	Estudo de coorte	2016	Brasil
24	*Incidence of phlebitis and post-infusion phlebitis in hospitalised adults*	Estudo de coorte	2017	Brasil
25	*Incidence and risk factors of phlebitis associated to peripheral intravenous catheters*	Estudo de coorte	2017	Espanha
26	*Observational Study of Peripheral Intravenous Catheter Outcomes in Adult Hospitalized Patients: A Multivariable Analysis of Peripheral Intravenous Catheter Failure*	Coorte prospectivo	2017	Austrália
27	*Dressings and securements for the prevention of peripheral intravenous catheter failure in adults (SAVE): a pragmatic, randomised controlled, superiority trial*	Ensaio clínico randomizado	2018	Austrália
28	*Incidence, severity and risk factors of peripheral intravenous cannula-induced complications: An observational prospective study*	Estudo observacional prospectivo	2018	Sérvia
29	*Prevalence of peripheral intravenous catheter-related phlebitis: associated factors*	Estudo de coorte prospectiva	2018	Portugal
30	*Phlebitis and infiltration: vascular trauma associated with the peripheral venous catheter*	Estudo de coorte descritiva	2018	Portugal
31	*Complicações locais da terapia intravenosa periférica e fatores associados*	Estudo Transversal	2018	Brasil
32	*Phlebitis-related peripheral venous catheterization and the associated risk factors*	Estudo prospectivo observacional	2018	Turquia
33	*Use of infrared thermography in the detection of superficial phlebitis in adult intensive care unit patients: A prospective single-center observational study*	Estudo observacional prospectivo	2019	Holanda
34	*A Model of Phlebitis Associated with Peripheral Intravenous Catheters in Orthopedic Inpatients*	Estudo prospectivo	2019	Coreia do Sul
35	*The RESPECT trial-Replacement of peripheral intravenous catheters according to clinical reasons or every 96 hours: A randomized, controlled, non-inferiority trial*	Ensaio randomizado multicêntrico, não cego e controlado	2020	Brasil
36	*In-line filtration reduced phlebitis associated with peripheral venous cannulation: Focus on cost-effectiveness and patients’ perspectives*	Estudo de análise econômica; ensaio clínico randomizado; custo-efetividade	2020	Itália
37	*Evaluation of the risk factors on time to phlebitis- and nonphlebitis-related failure when peripheral venous catheters were replaced as clinically indicated*	Estudo de coorte prospectiva	2020	Turquia
38	*Inherent and modifiable risk factors for peripheral venous catheter failure during cancer treatment: a prospective cohort study*	Estudo de coorte prospectivo	2021	Austrália
39	*Compare the efficacy of recommended peripheral intravascular cannula insertion practices with a standard protocol: A randomized control trial*	Ensaio clínico randomizado	2022	Índia
40	*Incidence, risk factors and medical cost of peripheral intravenous catheter-related complications in hospitalised adult patients*	Estudo prospectivo observacional	2022	China
41	*Risk factors for peripheral intravenous catheter-related phlebitis in adult patients*	Análise *post hoc* de um ensaio clínico randômico controlado	2022	Brasil
42	*A Prospective Sonographic Evaluation of Peripheral Intravenous Catheter Associated thrombophlebitis*	Estudo de coorte prospectivo	2022	EUA
43	*Risk factors for peripheral intravascular catheter-related phlebitis in critically ill patients: analysis of3429 catheters from 23 Japanese intensive care units*	Estudo de coorte prospectiva	2022	Japão
44	*Observational study of peripheral intravenous catheter outcomes in an internal medicine department*	Estudo prospectivo observacional	2022	Israel

A partir dos resultados constantes dos Quadros 2, 3 e 4, indica-se a prevalência dos estudos com nível de evidência IV (67,7%). Entre os principais desfechos, destacaram-se o tempo de permanência do cateter, uso de antibióticos e outros fármacos, contraste, material do cateter utilizado, *gauge* e número do cateter inserido. Houve variabilidade na forma de mensuração da flebite, com a aplicação de instrumentos distintos. Alguns estudos utilizaram os próprios sinais de flebite para realizar o diagnóstico.

**Quadro 2:** Quadro síntese dos estudos primários, incluídos na revisão integrativa conforme os fatores de risco associados à flebite relativos ao paciente e nível de evidência, São Paulo, São Paulo, Brasil, 2023

Fatores de risco associados à ocorrência de flebite: relacionados ao paciente	Nível de evidência
A presença de flebite foi estatisticamente significante quando associada a pacientes do sexo feminino^(26)^.	IV
Idade (18 a 47 anos), pacientes com mais de seis dias de internação e contagem de plaquetas (>303.000)^(15)^.	IV
Houve significância estatística referente à cor da pele (pele branca e parda associada à flebite grau I e pele negra associada à flebite grau III) e idade (o grupo de pacientes de 19 a 48 anos e o grupo de 71 a 95 anos se associaram à flebite grau I e grupo de pacientes de 49 a 70 anos se associou à flebite grau III)^(24)^.	IV
Presença de infecções e comorbidades aumentou em cerca de 1,5 vezes a chance de desenvolver flebite. Flebites grau III e IV foram diagnosticadas em pacientes com mais de 70 anos, desnutridos e com diabetes *mellitus* ^(28)^.	IV
Os CIPs tiveram uma probabilidade menor de apresentar flebite a cada aumento na idade (por década), se o paciente apresentasse três ou mais comorbidades^(38)^.	IV
Presença de infecção e doenças crônicas tiveram relação significativa com o desenvolvimento de flebite^(19)^.	IV
A flebite foi significativamente mais presente em CIP em pacientes com comorbidades, infecções atuais e com sonda vesical de demora^(28)^.	IV
Pouca elasticidade da pele (taxa de risco: 1,47; Intervalo de Confiança de 95%: 1,07–2,02; valor de *p* = 0,015)^(37)^.	IV
Houve relação significativa entre ter uma doença crônica e o desenvolvimento de flebite^(32)^.	IV
A flebite foi associada a pacientes ativos e qualidade da veia (regular e ruim)^(34)^.	IV
Internação na clínica neurológica (valor de *p* 0,018) foi comparada com internação na clínica médica^(31)^.	VI
Pacientes em clínica de neurocirurgia. Houve número maior de flebites em pacientes com doenças cranianas com CIP^(18)^.	VI
Pacientes em unidades cirúrgicas (pode estar associado à hipovolemia e ao longo período de uso do CIP)^(40)^.	IV
O acréscimo de um dia, para qualquer período de internação, aumentou a probabilidade do paciente apresentar flebite em 1,07 vezes^(30)^.	IV
Mobilidade reduzida, história familiar de trombose venosa profunda^(41)^.	II

*CIP - cateter intravenoso periférico.*

**Quadro 3:** Quadro síntese dos estudos primários, incluídos na revisão integrativa conforme os fatores de risco associados à flebite relativos à terapia intravenosa e nível de evidência, São Paulo, São Paulo, Brasil, 2023

Fatores de risco associados à ocorrência de flebite: relacionados à TIV	Nível de evidência
Tempo de permanência do CIP superior a 72 horas^(16)^.	IV
Tempo de permanência do CIP ≥72 horas, punção em antebraço e a infusão dos medicamentos como ceftriaxona (amostra = 7; 25%), claritromicina (amostra = 7; 28%) e oxacilina (amostra = 6; 46,2%)^(23)^.	IV
Tempo de permanência do cateter de 49-72 horas^(18)^.	VI
Tempo de permanência do CIP ≥ 3,25 dias^(29)^.	IV
Houve relação estatística significativa entre a duração do cateterismo (49-72 horas), o tipo de fluido utilizado (isotônico) e o desenvolvimento de flebite^(32)^.	IV
Tempo de permanência do CIP (91,5 horas) foi associado à ocorrência de flebite^(33)^.	IV
Tempo de permanência do cateter (25-48horas), método de infusão (contínuo e intermitente), uso de meio de contraste, medicamentos com alta osmolaridade^(34)^.	IV
Verificou-se que 9,0% dos pacientes desenvolveram flebite sem diferença entre os grupos. A substituição do CIP, quando clinicamente indicada, não foi inferior à substituição de rotina (96 horas) em relação à ocorrência de flebite, e foi associada II a significativamente menos flebites/1.000 cateter dia^(35)^.	II
Administração de menos que três medicações, administração de protetores de mucosa ou antissecretores gástricos e o uso de medicamentos antibacterianos^(15)^.	IV
Houve relação significativa entre flebite e tipo de infusão, e o uso associado de infusão contínua e intermitente teve uma relação maior com a ocorrência de flebite^(19)^.	VI
A flebite relacionada ao CIP pode ser significativamente associada à medicação intravenosa (infusão de aminoácidos), com remoção mais precoce prevista pelo local de inserção (articulação do cotovelo)^(20)^.	IV
Com relação à análise individual do uso de medicamentos, apresentaram significância estatística o cloridrato de tramadol, a amoxacilina + ácido clavulânico e a anfotericina (flebite pós-infusional). Na análise da associação das classes medicamentosas, apresentaram associação positiva os antifúngicos, os anti-inflamatórios e os que atuam no sangue e nos órgãos formadores de sangue (não especificados quais medicamentos)^(24)^.	IV
O uso de tubo de extensão como acessório do CIP^(25)^.	IV
Pacientes em uso de flucloxicilina intravenosa ou com CIP frequente^(26)^.	IV
Pacientes com administração, via CIP, de menos medicamentos (todos os grupos) e soluções de risco, com menos dias de administração de grupos de medicamentos, com e sem risco, e soluções de risco^(28)^.	IV
O uso do filtro em linha reduziu a incidência de flebite pós-operatória relacionada ao CIP e tem potencial para contribuir com o aumento da satisfação do paciente e reduzir o desconforto relacionado ao cateter^(36)^.	II
Uso de fármacos irritantes ao endotélio vascular^(40)^.	IV
CIP no dorso da mão e relato de dor, administração intravenosa com amoxicilina-clavulanato de potássio e omeprazol sódico^(41)^.	II
Uso de nitroglicerina (valor de *p* 0,04), nicardipina (valor de *p* 0,0008), noradrenalina (valor de *p* 0,002), amiodarona (valor de *p* 0,0006) e levetiracetam (valor de *p* < 0,0001)^(43)^.	IV
Uso de antibióticos intravenosos^(44)^.	IV

*TIV - terapia intravenosa; CIP - cateter intravenoso periférico.*

**Quadro 4:** Quadro síntese dos estudos primários, incluídos na revisão integrativa conforme os fatores de risco associados à flebite relativos ao procedimento e nível de evidência, São Paulo, São Paulo, Brasil, 2023

Fatores de risco associados à ocorrência de flebite: relacionados ao procedimento	Nível de evidência
Receber menos cuidados de enfermagem foi preditor de flebite relacionada ao CIP. Para cada aumento de unidade em cuidados perdidos, houve um aumento de 3,8% no risco de desenvolver flebite. A ausência de avaliação do local de inserção do CIP e outros cuidados de enfermagem atrasou o reconhecimento da flebite. Estar em um hospital especializado foi associado ao menor risco de flebite relacionada ao CIP^(17)^.	IV
Número maior de flebites em pacientes que tiveram CIP inseridos no mesmo local várias vezes e inseridos por profissionais com formação de quatro anos comparados aos que tinham formação de dois anos^(18)^.	VI
A flebite ocorreu com maior frequência quando a ponta do cateter foi colocada em ângulo >5,8°^(21)^.	IV
A flebite foi estatisticamente significante quando associada a hematomas no local de inserção do CIP e inserção no lado dominante dos pacientes^(26)^.	IV
Nenhuma diferença significativa na incidência de deslocamento e flebite foi identificada entre qualquer um dos grupos de intervenção em comparação com o grupo controle de poliuretano^(27)^.	II
A flebite foi mais presente em CIP de diâmetro 20 e 18 *gauge,* feitos de Teflon®, que ficaram *in situ* por mais de 96 horas. A chance é cerca de duas vezes maior no diâmetro do CIP de 22 e 20 *gauge,* e é quase três vezes maior no CIP de 18 gauge^(28)^.	IV
Ao aumentar um CIP para qualquer quantidade, aumentou-se a probabilidade em 1,37 vezes do paciente desenvolver flebite^(30)^.	IV
A análise multivariada identificou que a diferença de temperatura da pele (0,90°) está associada à ocorrência da flebite^(33)^.	IV
Duração da higiene das mãos (<10 segundos), período de experiência clínica de enfermagem (1-3 anos)^(34)^.	IV
Houve inserção malsucedida na primeira tentativa (taxa de risco: 1,35; Intervalo de Confiança 95%: 1,04-1,83; valor de *p* = 0,047) e uso de cateteres fabricados localmente (Teflon® fabricado na Turquia) (taxa de risco: 1,61; Intervalo de Confiança 95%: 1,18-2,20; valor de *p* = 0,002)^(37)^.	IV
Inserção na articulação do punho. Houve diminuição significativa na taxa de tromboflebite após a aplicação do protocolo padrão de limpeza do local de inserção em comparação à recomendação do Centro de Controle e Prevenção de Doenças. A incidência de tromboflebite aumentou significativamente mediante a segunda tentativa de inserção do CIP^(39)^.	II
O aumento da proporção do cateter em relação ao tamanho da veia (maior ou igual a 33,3%) e o ângulo mais acentuado da ponta do cateter (≥5 graus) aumentaram o risco de tromboflebite^(42)^.	IV

*CIP - cateter intravenoso periférico.*

## DISCUSSÃO

Nos últimos anos, houve um aumento do número de publicações sobre os fatores de risco associados à ocorrência de flebite em pacientes adultos hospitalizados que utilizaram CIP. Verificou-se, nesses estudos, variabilidade na forma de medição da ocorrência desse EA. Ressalta-se que, das 71 escalas de flebite em uso no mundo, apenas três passaram por alguma análise psicométrica, e a ausência de consenso sobre o uso de instrumentos de avaliação pode contribuir para as disparidades encontradas na literatura^(45)^. Outro fator dificultador é que alguns estudos^(16, 22, 28, 31, 37, 38, 40)^ analisaram a ocorrência de flebite, infiltração, extravasamento e oclusão conjuntamente, e os fatores de risco foram, de forma geral, relacionados às falhas relativas ao CIP, consistindo variáveis que podem gerar confusão entre os fatores de risco.

Apesar de estudos^(14, 18, 23, 28, 29, 32, 34)^ indicarem que o tempo de permanência do CIP é um fator de risco à flebite, revisão sistemática^(46)^, ao avaliar os efeitos da troca de CIP, quando clinicamente indicada, comparada com a troca rotineiramente programada, a partir de uma amostra de sete ensaios clínicos randomizados com 4.895 pacientes, verificou que não houve diferença entre esses grupos em nenhuma das medidas analisadas e que os custos médios foram menores quando os CIPs foram substituídos de acordo com a indicação clínica. O estudo indicou, ainda, que a troca rotineira do CIP pode gerar dor, desconforto e insatisfação do paciente devido às novas punções^(46)^.

O material do cateter utilizado pode estar associado à flebite^(23, 28, 37)^. o dispositivo de poliuretano possui maior flexibilidade, quando comparado ao de Teflon®, minimizando os danos causados à parede vascular e desempenhando um papel importante na minimização do desenvolvimento desse EA^(21)^. Os cateteres flexíveis, como os de poliuretano, estão associados a menores complicações e a redução de flebites^(47)^. Além disso, recomenda-se a avaliação do vaso para que o cateter utilizado seja do tamanho adequado^(5)^. CIPs com *gauge* maior que 20 estão mais associados com a ocorrência de flebite^(5, 25, 28)^.

É importante considerar os pacientes com acesso venoso difícil (*difficult intravenous access* (DIVA)), com duas ou mais tentativas malsucedidas de punção intravenosa periférica, usando a técnica tradicional, além de ausência de veias visíveis ou palpáveis ou paciente com história relatada ou documentada de DIVA^(48)^. Esses pacientes precisam ser avaliados, antes da punção, para evitar múltiplas tentativas, visto que a quantidade de tentativas está relacionada a falhas no CIP^(18, 28, 30, 37, 39)^.

Ao reconhecer que as tentativas malsucedidas causam danos, a *Infusion Nurses Society* (INS) recomenda que, após duas tentativas malsucedidas, é preciso direcionar o procedimento para um profissional com nível de habilidade mais alto ou o uso de vias alternativas de administração^(5)^.

A presente revisão evidenciou a associação de alguns fármacos à ocorrência de flebite^(18, 23, 26, 27, 29, 37, 41, 43, 44, 46, 47)^, como ceftriaxona, clindamicina, oxacilina, contraste, nitroglicerina, nicardipina, noradrenalina, amiodarona, anfotericina e cloridrato de tramadol. Nessa direção, enfatiza-se que o planejamento seguro da TIV, minimizando os possíveis riscos, não pode prescindir do adequado conhecimento dos fármacos que são danosos à rede venosa^(49)^.

Estudo espanhol, realizado por uma equipe de médicos, enfermeiras e farmacêuticos, padronizou as diluições dos medicamentos não oncológicos mais utilizados em pacientes adultos hospitalizados. A lista de medicamentos foi estabelecida por meio de consenso visando reduzir a variabilidade do cuidado, melhorar a segurança na TIV e sugerir diferentes níveis de risco. Conhecer os medicamentos e suas características contribui para a escolha do dispositivo mais adequado para o paciente, visando reduzir a ocorrência de complicações como a flebite^(49)^.

Os cuidados de enfermagem são essenciais para o gerenciamento do CIP e minimizar a ocorrência de EA; cuidados de enfermagem perdidos afetam a incidência de flebite^(34)^; a ausência de avaliação do local de inserção do CIP atrasa o reconhecimento da flebite^(17)^. Ademais, durante a avaliação, o paciente pode relatar presença de dor, sintoma que pode estar associado à flebite^(41)^.

A presença de infecção^(19, 28)^ e doenças crônicas^(19, 28, 32, 38)^ esteve presente como fator de risco para flebite. Logo, o enfermeiro precisa conhecê-los para prestar cuidado individualizado a esses pacientes, os quais podem permanecer hospitalizados por um período maior, e a internação prolongada aumenta o risco do desenvolvimento de flebite^(15, 30)^.

Os resultados obtidos nesta revisão são semelhantes aos da anteriormente citada^(6)^, notadamente com relação à diversidade na forma como a flebite é mensurada, o que indica a necessidade de outras investigações sobre a grande variabilidade dos instrumentos utilizados, sendo preciso um alinhamento entre os pesquisadores dessa temática. Outros fatores como tempo de permanência do CIP, local de punção, tempo de internação do paciente, quantidade de acessos e uso de antibióticos foram semelhantes aos da revisão supramencionada.

Destaca-se que a presente revisão avança com relação ao conhecimento existente, ao evidenciar que receber menos cuidados de enfermagem, mobilidade reduzida do paciente, história familiar de trombose venosa profunda, relato de dor na inserção do CIP, qualidade da veia do paciente, elasticidade da pele e inserção do CIP malsucedida estão associados à ocorrência de flebite.

Visando subsidiar a tomada de decisão, previamente à punção intravenosa periférica, a fim de contribuir para a preservação da saúde vascular do paciente, corrobora-se, como aspecto a ser considerado em pesquisas futuras, a identificação dos pacientes com DIVA e a sua associação com a ocorrência da flebite^(50)^. Ressalta-se que, após duas tentativas malsucedidas para obtenção do acesso via CIP, a INS recomenda que seja acionado um profissional com maior nível de habilidade e suporte tecnológico para realizar o procedimento, além de considerar outra via para administração do fármaco^(5)^. Então, recomenda-se a condução de estudos com maior nível de evidência que avaliem a saúde vascular dos pacientes, a realização de programas de capacitação específicos por enfermeiros, a individualização do cuidado prestado, a punção venosa periférica guiada por ultrassom e os desfechos associados, e a identificação precoce dos pacientes com DIVA, possibilitando, além de evitar complicações, a elaboração de diretrizes clínicas quanto aos cuidados requeridos.

### Limitações do estudo

Apenas a inclusão de artigos de acesso gratuito, disponíveis na íntegra nas bases de dados estabelecidas, pode ser indicada como uma limitação deste estudo.

### Contribuições para a área da enfermagem, saúde, ou políticas públicas

Por intermédio da presente revisão integrativa, corroboraram-se fatores de riscos já evidenciados na literatura, e identificaram-se novos fatores associados ao desenvolvimento de flebite em pacientes adultos hospitalizados, notadamente o reconhecimento de pacientes com DIVA. Destaca-se, a partir do conhecimento gerado, a pertinência da abordagem proativa para preservar os vasos para necessidades futuras, melhorar a execução do plano de cuidado, reduzir o tempo de internação e, consequentemente, os custos associados à assistência prestada.

## CONCLUSÕES

Nesta revisão integrativa, foram analisados 31 (100,0%) estudos primários quantitativos sobre os fatores de risco associados à ocorrência de flebite em pacientes adultos hospitalizados que utilizaram CIP, com predomínio dos estudos com nível de evidência IV (67,7%), do tipo coorte (45,1%) e desenvolvidos no Brasil (25,8%).

Foram sintetizados os seguintes fatores de risco: tempo de internação; alteração hematológica; número de medicamentos administrados; uso de antibióticos; tempo de permanência do CIP; receber menos cuidados de enfermagem; cateter inserido várias vezes; pacientes com infecção e comorbidades; presença de dor no local de inserção do CIP; uso do cateter de Teflon®; mobilidade reduzida do paciente; história familiar de trombose venosa profunda; qualidade da veia do paciente; elasticidade da pele; e inserção malsucedida. Ressalta-se a imprescindibilidade de uniformizar o formato de medição da ocorrência desse EA e desenvolver novos estudos com maior nível de evidência.
